# Occult Metastatic Invasive Lobular Breast Carcinoma Presenting as Gastric Outlet Obstruction and Peritoneal Carcinomatosis

**DOI:** 10.7759/cureus.114163

**Published:** 2026-08-08

**Authors:** Kristin Pekmezian, Sara Ubosy, Mustafa Nuaimi, Jorge Parellada, Steve Carlan

**Affiliations:** 1 Internal Medicine, Orlando Regional Medical Center, Orlando, USA; 2 Obstetrics, Orlando Regional Medical Center, Orlando, USA

**Keywords:** breast lobular carcinoma, disseminated carcinomatosis, gastric outlet obstruction(goo), invasive carcinoma, primary breast neoplasm

## Abstract

Invasive lobular carcinoma (ILC) of the breast is a common type of breast cancer and is known to have a distinct metastatic pattern, with a predilection for the gastrointestinal tract and peritoneum rather than the lungs and liver. Gastric outlet obstruction (GOO), as the initial manifestation of occult metastatic breast carcinoma, is rare and may closely mimic primary gastrointestinal malignancy.

A 67-year-old woman presented with progressive nausea, vomiting, poor oral intake, weight loss, and symptoms consistent with GOO. Computed tomography demonstrated gastric wall thickening, ascites, and peritoneal abnormalities concerning for primary gastric malignancy. Initial endoscopic biopsies of the pyloric channel revealed erosive chemical gastropathy without evidence of malignancy. Given the high clinical suspicion, endoscopic ultrasound-guided fine-needle aspiration (EUS-FNA) was performed, which demonstrated adenocarcinoma. Immunohistochemistry showed positivity for CK7, GATA3, and TRPS1, with negativity for CK20 and CDX2, favoring a breast primary. Subsequent diagnostic laparoscopy with omental, peritoneal, and mesenteric biopsies confirmed metastatic carcinoma with attenuated E-cadherin expression, consistent with invasive lobular breast carcinoma. Despite extensive biopsy-proven peritoneal disease, peritoneal fluid cytology was negative for malignant cells. Biomarker analysis demonstrated estrogen receptor positivity (91%-100%), progesterone receptor positivity, HER2 negativity (1+), and a Ki-67 of 10%-15%.

This case highlights several diagnostic challenges in metastatic ILC, including false-negative mucosal biopsies, negative peritoneal cytology despite extensive carcinomatosis, and the need for deeper tissue sampling. Recognition of contemporary immunophenotypic markers, including GATA3 and TRPS1, with attenuated E-cadherin expression, is essential for distinguishing metastatic breast carcinoma from primary gastrointestinal malignancies and for facilitating appropriate systemic therapy.

## Introduction

Invasive lobular carcinoma (ILC) is the second most common histologic subtype of breast cancer, accounting for approximately 10%-15% of invasive breast malignancies [1]. Unlike invasive ductal carcinoma (IDC), ILC has a distinctive metastatic pattern, with increased involvement of the gastrointestinal tract, peritoneum, retroperitoneum, ovaries, and leptomeninges [2,3]. Loss of E-cadherin disrupts cell adhesion and contributes to the diffuse infiltrative growth pattern characteristic of lobular carcinoma, likely explaining its distinctive metastatic behavior [4].

Metastatic involvement of the stomach by breast carcinoma is uncommon and often mimics primary gastric adenocarcinoma clinically, radiographically, endoscopically, and histologically [5]. Patients may present with nonspecific symptoms, including abdominal pain, nausea, vomiting, early satiety, weight loss, and, rarely, gastric outlet obstruction (GOO) [6].

Although several case reports have described GOO secondary to metastatic lobular breast carcinoma, many involved patients with previously diagnosed breast cancer or with a diagnosis established by conventional gastric biopsies [7,8]. We report a case of occult hormone receptor-positive, HER2-negative ILC presenting with GOO and diffuse peritoneal carcinomatosis, with initially negative gastric biopsies. Initial gastric biopsies and peritoneal cytology were negative, and the diagnosis required EUS-guided sampling and laparoscopic tissue biopsy.

## Case presentation

A 67-year-old woman presented with progressive nausea, recurrent vomiting, poor oral intake, abdominal discomfort, and significant unintentional weight loss. Laboratory studies showed hypokalemia, leukocytosis, and mild normocytic anemia (Table 1).

**Table 1:** Pertinent lab values on admission.

Variable	Normal Lab Values	Admission
Hemoglobin (g/dL) (grams/deciliter)	12.3 to 15.3 gm/dL	9.1
White blood cell (cells/µL) (cells/microliter)	4,500 - 11,000 cells/µL	13,000
Mean corpuscular volume (fL) (femtoliters)	80 - 100 femtoliters (fL)	87
Potassium (mEq/L) (milliequivalents per liter)	3.5 - 5.5 mEq/L	2.9

**Figure 1:**
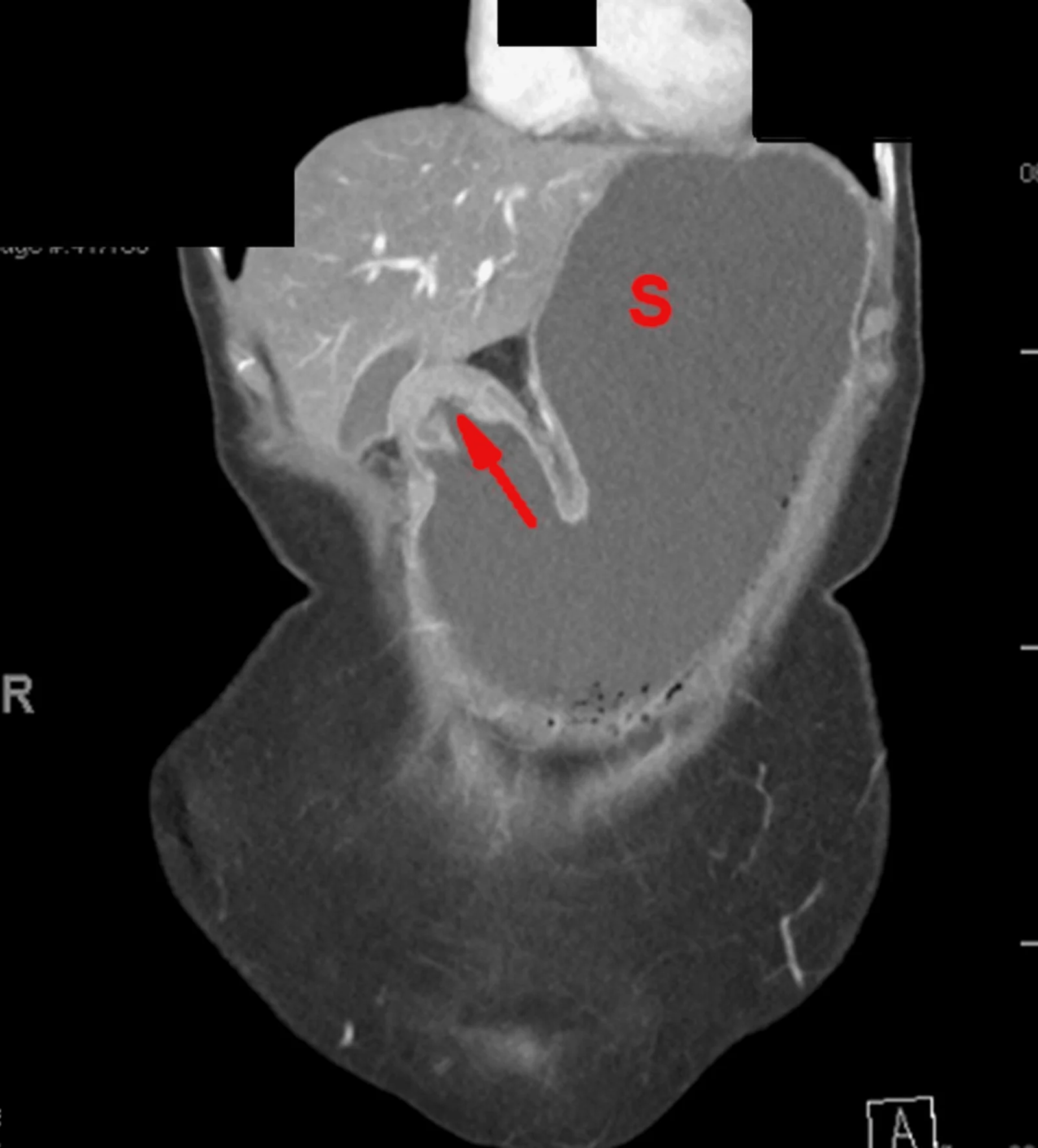
CT with contrast of the abdomen and pelvis. ‘S’ denotes the enlarged stomach. The arrow shows the region of outlet obstruction.

Computed tomography of the abdomen and pelvis revealed abnormalities of the distal stomach with associated ascites and diffuse peritoneal abnormalities, concerning for a primary gastric malignancy causing GOO (Figure 1).

**Figure 2:**
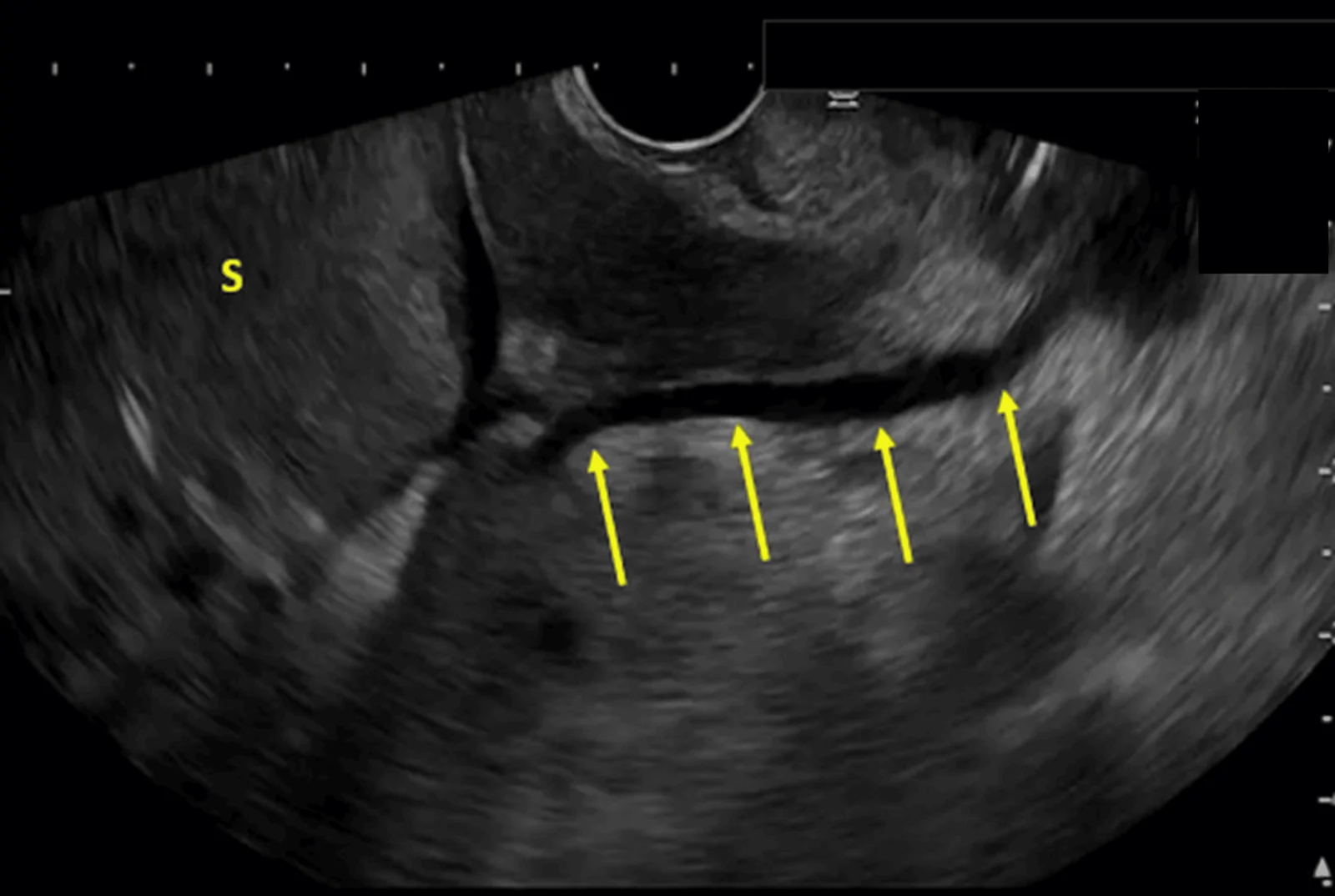
Radial endoscopic ultrasound (EUS) scan highlighting a narrow, elongated hypoechoic channel. The yellow arrows point to a markedly thickened pyloric muscle layer surrounding a compressed pyloric canal. ‘S’ is the stomach.

**Figure 3:**
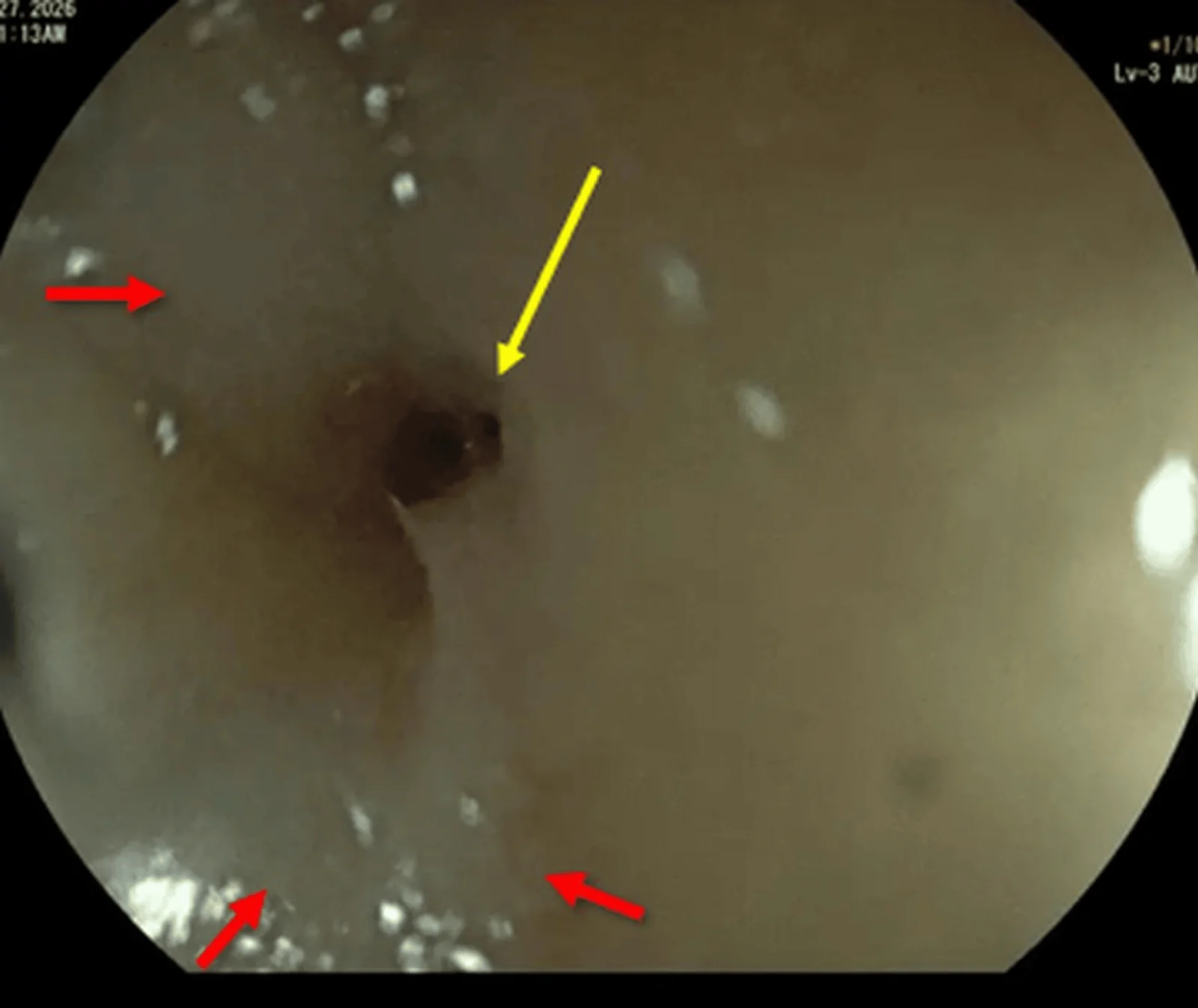
The image shows a highly narrowed, pinhole-sized opening at the pylorus (yellow arrow), which prevents a standard upper endoscope from passing through into the duodenum. The surrounding mucosa appears somewhat thickened or edematous (red arrows), consistent with structural obstruction or narrowing.

Given persistent obstructive symptoms, endoscopic ultrasound (EUS) (Figure 2) and esophagogastroduodenoscopy (EGD) (Figure 3) were performed.

Biopsies from the pyloric channel showed fragments of gastric body and antral-type mucosa with erosive chemical gastropathy. No intestinal metaplasia, dysplasia, *Helicobacter pylori* infection, or malignancy was identified. Despite these negative findings, the radiographic appearance and clinical presentation remained highly suspicious for an infiltrative malignant process.

**Figure 4:**
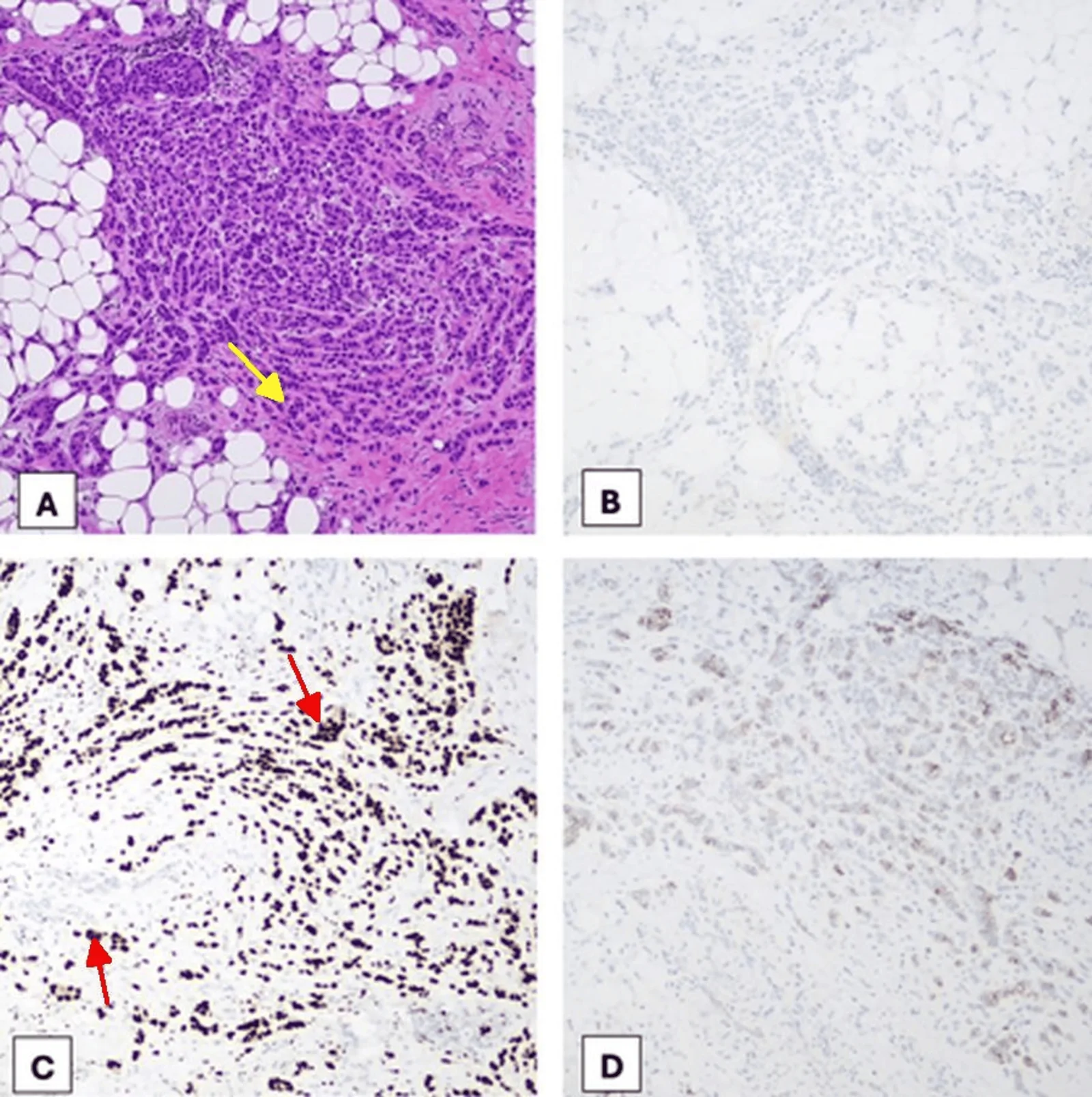
(A) Hematoxylin and eosin-stained sections of the omentum show tumor cells infiltrating the fibroadipose tissue in cords and single cells (yellow arrow). The cells are small and uniform, with scant cytoplasm, lacking marked atypia, 100×. (B) This image presents an immunohistochemical (IHC) assay (CDX2) yielding a completely negative result. The clear-to-light-blue appearance indicates that the targeted protein biomarker is absent in these specific cells, 400×. (C) This image displays a highly positive IHC stain, characterized by a strong 3+ brown signal. The red arrows point to regions with prominent, dense staining, characteristic of GATA3 positivity, 400×. (D) The tumor cells exhibit attenuated expression of E-cadherin, 400×. The pattern and localization of E-cadherin staining are qualitatively assessed.

Given persistent concern for malignancy, EUS-guided fine-needle aspiration (FNA) of a gastric lesion was performed four days later. Cytologic examination demonstrated adenocarcinoma. Immunohistochemical analysis showed positivity for cytokeratin AE1/AE3, CK7, GATA3, and TRPS1, with negative staining for CK20 and CDX2. Although poorly cohesive gastric adenocarcinoma was considered in the differential diagnosis, the immunophenotype favored metastatic breast carcinoma. Given concern for disseminated intra-abdominal disease, the patient subsequently underwent diagnostic laparoscopy 10 days after the diagnosis. Biopsies from the omentum, left lower quadrant peritoneum, and a small bowel mesenteric nodule demonstrated metastatic carcinoma (Figure 4).

Histopathologic evaluation of the omental specimen showed tumor cells positive for CK7, GATA3, and TRPS1 and negative for CK20 and CDX2. E-cadherin expression was attenuated, a finding characteristic of ILC. Peritoneal and mesenteric biopsies showed similar metastatic involvement, with GATA3 positivity and CDX2 negativity.

Interestingly, peritoneal fluid cytology obtained during laparoscopy was negative for malignant cells and showed only reactive mesothelial cells, histiocytes, and lymphocytes, despite extensive biopsy-confirmed peritoneal carcinomatosis.

Breast biomarker analysis of both gastric and omental specimens showed strong estrogen receptor expression (91%-100%, 3+), progesterone receptor positivity (21%-30% in the gastric specimen and 81%-90% in the omental specimen, both 3+), HER2 negativity (IHC score 1+), and a Ki-67 proliferation index of 10%-15%. PD-L1 (22C3) testing was negative. Molecular profiling via Caris testing was initiated.

Peripheral blood smear evaluation demonstrated marked leukocytosis with left-shifted neutrophilia without circulating blasts or overt dysplasia, findings consistent with a reactive process. Mild normocytic anemia with anisopoikilocytosis was also present.

Collectively, the clinicopathologic findings established the diagnosis of stage IV hormone receptor-positive, HER2-negative metastatic invasive lobular breast carcinoma presenting with GOO and diffuse peritoneal carcinomatosis. She underwent laparoscopically assisted gastrostomy tube placement to relieve the obstruction and laparoscopically assisted jejunostomy for feeding. She was also started on total parenteral nutrition (TPN). Given the poor prognosis, the plan was to discuss palliative interventions and potentially initiate paclitaxel once she was more stable and better conditioned. A CDK4/6 inhibitor was considered unsuitable because of unpredictable enteral absorption secondary to TPN dependence, jejunostomy, and possible visceral crisis.

## Discussion

ILC accounts for approximately 10%-15% of breast cancers and has biological and metastatic features distinct from those of IDC. Loss of E-cadherin-mediated cell adhesion leads to a diffuse infiltrative growth pattern, and ILC preferentially metastasizes to unusual sites, including the gastrointestinal tract, peritoneum, retroperitoneum, ovaries, and leptomeninges, rather than the lungs and liver, which are commonly involved in IDC [1-4].

Gastrointestinal metastases from breast carcinoma are uncommon and often mimic primary gastrointestinal malignancies on clinical, radiographic, endoscopic, and histologic evaluation [5,6]. Patients may present with nonspecific symptoms such as nausea, vomiting, abdominal pain, early satiety, weight loss, gastrointestinal bleeding, or GOO. Malignancy is the most common cause of GOO, most often arising from primary gastric or pancreatic cancers, whereas metastatic breast carcinoma is a rare cause [6].

Several cases of GOO secondary to metastatic lobular breast carcinoma have been reported [7,8]. In most reports, patients had a known history of breast cancer before the onset of gastrointestinal symptoms [7,8]. Cases of occult primary ILC with gastrointestinal and peritoneal involvement have also been described, though without presenting as GOO [9]. In contrast, our patient had no prior history of breast malignancy, and GOO was the initial manifestation of disseminated disease.

An important diagnostic feature of this case was the discrepancy between negative mucosal biopsies and subsequent positive EUS-guided tissue acquisition. Initial pyloric biopsies showed only erosive chemical gastropathy despite extensive underlying malignancy. This pattern reflects the known tendency of metastatic ILC to infiltrate the submucosa and muscularis propria while sparing the superficial mucosa, resulting in a high rate of false-negative endoscopic biopsies [5,8]. Consequently, deeper sampling is essential when clinical suspicion remains high. In our patient, EUS-guided FNA was critical in establishing the diagnosis, followed by laparoscopic biopsy given extensive peritoneal disease.

Another notable finding was biopsy-confirmed peritoneal carcinomatosis despite negative peritoneal fluid cytology. Cytologic analysis showed only reactive mesothelial cells, histiocytes, and lymphocytes. This underscores that negative cytology does not exclude peritoneal metastatic disease, and histologic confirmation remains the diagnostic gold standard when imaging and clinical findings are discordant.

Immunohistochemistry was essential for distinguishing metastatic breast carcinoma from primary gastrointestinal adenocarcinoma. Previous studies have demonstrated the diagnostic utility of breast-associated markers, including estrogen receptor, progesterone receptor, GCDFP-15, mammaglobin, and GATA3, in establishing mammary origin [10]. In our case, the tumor was positive for CK7, GATA3, and TRPS1, with negative CK20 and CDX2 staining and attenuated E-cadherin expression, findings that strongly support a diagnosis of metastatic ILC. GATA3 is a highly sensitive marker for breast carcinoma, while TRPS1 has recently emerged as a highly sensitive and specific marker with increasing use in contemporary breast pathology [10,11]. Together with loss of E-cadherin expression, these findings allowed reliable distinction from primary gastric adenocarcinoma and supported the diagnosis of metastatic invasive lobular breast carcinoma [4,10,11].

The tumor exhibited a luminal phenotype, characterized by strong estrogen receptor expression, progesterone receptor positivity, HER2 negativity, and a low proliferative index. These features are associated with favorable responses to endocrine therapy combined with CDK4/6 inhibitors, the preferred first-line treatment for hormone receptor-positive metastatic breast cancer [12,13].

Compared with previously published reports, our case uniquely combines several uncommon features: occult breast cancer without a prior diagnosis, GOO as the initial presentation, false-negative gastric mucosal biopsies, diagnosis established by EUS-guided and surgical tissue acquisition, biopsy-proven peritoneal carcinomatosis despite negative cytology, and confirmation with contemporary markers, including TRPS1 and attenuated E-cadherin expression (Table 2).

**Table 2:** Comparisons of previously published cases. ILC: invasive lobular carcinoma; GOO: gastric outlet obstruction; EUS-FNA: endoscopic ultrasound fine-needle aspiration; GI: gastrointestinal

Feature	J Med Case Rep 2012 [7]	Clinical Case Reports 2022 [8]	Cureus 2024 [9]	Present Case
Prior history of breast cancer	Yes	Yes (4 years prior)	No	No
Occult primary breast cancer	No	No	Yes	Yes
Gastric outlet obstruction	Yes	Yes	No	Yes
Peritoneal carcinomatosis	Not reported	No	Yes	Yes
Ascites	Not reported	No	Yes	Yes
Ovarian metastases	No	No	Yes	No
Initial gastric biopsy negative	No	Yes	No	Yes
EUS-guided diagnosis required	No	Yes	No	Yes
Additional laparoscopic biopsy required	No	No	No	Yes
Negative peritoneal cytology despite metastatic disease	Not reported	Not applicable	Not reported	Yes
GATA3 positive	Yes	Yes	Yes	Yes
TRPS1 performed	No	No	No	Yes
Attenuated E-cadherin demonstrated	Not reported	Not reported	Yes	Yes
ER+/HER2- phenotype	Yes	Yes	Yes	Yes
Major teaching point	Broaden GOO differential	Importance of EUS-FNA	Occult ILC with GI spread	Multimodal diagnosis required; cytology and superficial biopsies may be falsely negative

Several aspects of this presentation were notable. GOO was the initial manifestation of previously occult breast cancer. Initial gastric mucosal biopsies were nondiagnostic despite extensive disease, and definitive diagnosis required EUS-guided tissue acquisition followed by laparoscopic biopsy. Furthermore, extensive biopsy-proven peritoneal metastases were present despite negative peritoneal fluid cytology. The diagnosis was supported by contemporary immunohistochemical markers, including TRPS1 positivity and attenuated E-cadherin expression, findings characteristic of invasive lobular breast carcinoma.

One weakness of the paper was the wide gap in progesterone receptor positivity between the gastric specimen (21%-30%) and the omental specimen (81%-90%), both 3+. The reason for this discrepancy was not readily apparent but could have been secondary to tumor heterogeneity, pre-analytical tissue artifacts, analytical or sampling variations, or other less common causes [14].

## Conclusions

Primary invasive lobular breast carcinoma should be considered in the differential diagnosis of unexplained GOO and peritoneal disease, even in the absence of a known breast primary. Negative mucosal biopsies do not exclude malignancy because metastatic ILC frequently infiltrates the deeper layers of the gastrointestinal wall. This case highlights the diagnostic value of EUS-guided tissue acquisition, the limitations of peritoneal fluid cytology, and the importance of contemporary immunohistochemical markers - including GATA3, TRPS1, and attenuated E-cadherin expression - in distinguishing metastatic breast carcinoma from primary gastrointestinal malignancies. Early recognition of these features may facilitate timely diagnosis and appropriate systemic therapy.
